# The Diversity of Endophytic Methylotrophic Bacteria in an Oil-Contaminated and an Oil-Free Mangrove Ecosystem and Their Tolerance to Heavy Metals

**DOI:** 10.1155/2012/759865

**Published:** 2012-03-07

**Authors:** Manuella Nobrega Dourado, Anderson Ferreira, Welington Luiz Araújo, João Lúcio Azevedo, Paulo Teixeira Lacava

**Affiliations:** ^1^Department of Genetics, “Luiz de Queiroz” College of Agriculture, University of São Paulo, Avenida Pádua Dias 11, P.O. Box 83, 13400-970 Piracicaba, SP, Brazil; ^2^Brazilian Agricultural Research Corporation, Embrapa Agrosilvopasture, Avenida dos Jacarandás, 2639 Sinop, MT, Brazil; ^3^Department of Microbiology, Biomedical Sciences Institute, University of São Paulo, 05508-900 São Paulo, SP, Brazil; ^4^Institute of Natural Sciences, Federal University of Alfenas, Gabriel Monteiro da Silva Street 700, 37130-000 Alfenas, MG, Brazil

## Abstract

*Methylobacterium* strains were isolated from mangrove samples collected in Bertioga, SP, Brazil, from locations either contaminated or uncontaminated by oil spills. The tolerances of the strains to different heavy metals were assessed by exposing them to different concentrations of cadmium, lead, and arsenic (0.1 mM, 0.5 mM, 1 mM, 2 mM, 4 mM, and 8 mM). Additionally, the genetic diversity of *Methylobacterium* spp. was determined by sequence analysis of the 16S rRNA genes. The isolates from the contaminated locations were grouped, suggesting that oil can select for microorganisms that tolerate oil components and can change the methylotrophic bacterial community. Cadmium is the most toxic heavy metal assessed in this work, followed by arsenic and lead, and two isolates of *Methylobacterium* were found to be tolerant to all three metals. These isolates have the potential to bioremediate mangrove environments contaminated by oil spills by immobilizing the heavy metals present in the oil.

## 1. Introduction

Mangrove ecosystems are widely distributed, covering approximately from 60 to 75% of the world's coasts. These ecosystems are very important due to their great diversity of animals, plants, and microorganisms, and because they are some of the most productive environments in the world [1]. This diversity demands a high nutrient availability at the beginning of the trophic chain, conferring a high importance on the activities of microorganisms, such as bacteria, that are responsible for the processes of degradation and formation of essential compounds and for most of the carbon flow in the sediments of the mangrove forest [1]. The description and the distribution of the bacterial diversity in a mangrove forest allow for a better understanding of bacterial functions and their interactions in this ecosystem. The adaptation of bacterial species to mangrove conditions indicates a potential source of biotechnological resources to be explored, including the discovery of new bacterial species that produce enzymes that can be used for human life, agriculture, or industry [2]. 

The bacteria of the *Methylobacterium* genus belong to a subclass *α*-Proteobacteria that are able to degrade one-carbon compounds (C1) such as methanol and methylamine. Members of this genus are widely distributed in the environment, colonizing air, soil, sediment, water, plant nodules and grains (endophytically), and leaf surfaces (epiphytically) [3, 4]. *M. extorquens *is able to produce polyhydroxybutyrate (PHB) from methanol, and this process was described as an alternative to biodegradable plastic production [5]. Methylotrophic bacteria have an important role in the degradation of different toxic compounds such as phenol [6], monomethyl isophthalate and dimethyl isophthalate [7, 8], CH_3_Cl, a compound that damages the stratospheric ozone layer [9], and methyl tert-butyl ether (MTBE), which is widely used as a fuel oxygenate and has replaced tetraethyl lead in improving the combustion efficiency of gasoline [10–12]. Additionally, it is a potential sulfentrazone degrader [13].

In addition to its degradation of toxic compounds, *Methylobacterium *also tolerates heavy metals. *Methylobacterium* isolates found on the rhizosphere and endosphere of the hyperaccumulating plant *Thlaspi goesingense* exhibited multiple heavy metal resistances, including toward nickel, cadmium, copper, zinc, and chrome [14]. De Marco et al. [15] collected isolates from a range of soil and sediment sources that were resistant to extremely high concentrations of the heavy metals cadmium, chromium, mercury, lead, and the metalloid arsenic. *Methylobacterium* also reduces the toxicity of nickel and cadmium in plants, promotes plant growth [16], and colonizes and reduces the phytotoxicity of grass fibers [17].

The adaptation of the endophytic bacterial species to mangrove conditions reveals a potential source of biotechnological resources that deserve exploration, including the search for new bacterial species of environmental importance or for isolates with potential bioremediation applications for polluted environments. Moreover, comparisons of the descriptions and the distributions of the endophytic *Methylobacterium* diversity in mangrove forests with and without oil contamination will lead to a better understanding of the function of these bacteria and their interactions in this ecosystem. In this context, the aims of this study were twofold: (i) to detect endophytic bacteria of the *Methylobacterium* genus tolerant to heavy metals in three mangrove plant species and (ii) to identify and assess the genetic diversity of these isolates by 16S rRNA sequencing. 

## 2. Material and Methods

### 2.1. Mangrove Forest Plant Sampling

The mangrove forest samples were collected at Bertioga, São Paulo State, Brazil (23°51′16′′S/46°08′19′′W). These mangroves receive mixed water from the sea and the Iriri River. Two locations and three mangrove species were assessed: (A) a location contaminated by an oil spill and (B) an uncontaminated location; (1) *Rhizophora mangle*; (2) *Laguncularia racemosa*; (3) *Avicennia *spp. mangrove species, respectively. The sampling was performed in July 2008 (average temperature 18°C), with expeditions during the low tides. This mangrove forest suffered its most recent oil spill in 1983, when 35 million liters of oil spilled into the mangrove area; however, effects of anthropogenic impacts (domestic and industrial sewers) persist at both the contaminated and the uncontaminated sites. According to chemical analyses performed by Nishio et al., [18] the oil-contaminated mangrove swamp exhibits a lower humidity and higher amounts of sulfur than does the uncontaminated location. Moreover, the native vegetation is yet to completely recover. 

### 2.2. Isolation of Endophytic Bacteria from Branches

Plants were sampled twice, and the total endophytic bacterial populations were isolated from surface-disinfected branches (70% ethanol for 1 min, sodium hypochlorite solution (2% available Cl^−^) for 2 min, 70% ethanol for 1 min, and two washes in sterilized distilled water). After surface disinfection, the branches were triturated in sterile phosphate-buffered saline (PBS, containing (g L^−1^) Na_2_HPO_4_, 1.44; KH_2_PO_4_, 0.24; KCl, 0.20; NaCl, 8.00; pH 7.4) and maintained at 28°C with 150 r.p.m. agitation for 1 hour. Appropriate dilutions were subsequently plated onto 10% trypticase soy agar (TSA-Merck) supplemented with 50 mg/mL of benomyl fungicide to prevent fungal growth. The plates were incubated at 28°C for 10 days, and the number of colony forming units (CFU) was determined to estimate the population density of pink-pigmented bacteria. The efficiency of the disinfection process was verified by plating aliquots of the final sterile-distilled water wash onto 10% TSA and incubating them under the same conditions as described above. Following the isolation of the pink-pigmented colonies, single colonies were suspended in 20% glycerol solution and stored at −70°C.

### 2.3. PCR Detection of the *Methylobacterium* Genus

The pink-pigmented isolates were subjected to PCR using specific primers to detect the 16S rRNA gene in the *Methylobacterium* genus. These primers amplify an internal fragment of 250 bp in the 16S rRNA gene and were used as described by Ferreira et al. [19]. The following primer sequences were used for 16S rRNA gene amplification: 2F (5′-GATCGGCCCGCGTCTGATTAG-3′) and 2R (5′-CCGTCATTATCGTCCCGGACA-3′).

### 2.4. Molecular Identification and Diversity Analysis of *Methylobacterium* Strains

The 16S rRNA gene was amplified using colony PCR [20]. For the PCR, isolates were grown on TSA and picked colonies were transferred to tubes containing 200 *μ*L of sterilized ultrapure water. The bacterial suspensions were used as the DNA templates for PCR reactions. The following primer sequences were used for 16S rRNA gene amplification: PO27F (50-GAGAGTTTGATCCTGGCTCAG-30) and 1387R (50-CGGTGTGTACAAGGCCCGGGAACG-30). The PCR reaction mixture included 1 *μ*L of bacterial suspension, 10 mM Tris-HCl (pH 8.3), 3.75 mM MgCl_2_, 0.2 mM of each dNTP, 200 mM of each primer, and 2.5 U of Taq DNA polymerase (Fermentas Life Sciences, Brazil) in a 50 *μ*L final volume. A negative control (PCR mixture without DNA) was included in all PCR amplifications. Amplifications were performed in a thermal cycler with the following PCR parameters: initial denaturation at 94°C for 5 min, 35 cycles of denaturation at 94°C for 1 min, primer annealing at 62.5°C for 1 min, and primer extension at 72°C for 1 min, followed by a final extension at 72°C for 7 min. PCR products were analyzed by electrophoresis in a 1.2% (w/v) agarose gel stained with ethidium bromide in 0.5x TBE buffer.

For bacterial species identification, the 16S rRNA gene PCR products were purified with polyethylene glycol (PEG) (20% PEG 8000; 2.5 mM NaCl) and sequenced in an ABI 377 DNA Sequencer (ABI, USA). The sequences were compared with those deposited in Ribosomal Database Project (RDP database). The most similar sequences were retrieved and aligned to produce dendrograms using the MEGA software (Molecular Evolutionary Genetics Analysis, version 4) [21]. 

### 2.5. Tolerance to Heavy Metals

Pink-pigmented isolates were assessed for growth on solid CHOI 3 medium containing (NH_4_)_2_SO_4_, KH_2_PO_4_, Na_2_HPO_4_·7H_2_O, MgSO_4_·7H_2_O, trace metals, supplemented with methanol as carbon source [22] with the addition of the three different heavy metal salts: arsenic (As_2_O_5_), cadmium (CdCl_2_), and lead (PbCO_3_), at six different concentrations (0.1 mM, 0.5 mM, 1 mM, 2 mM, 4 mM, and 8 mM) independently dissolved in the culture medium. There were three replicates for each concentration and the plates were incubated for ten days at 28°C. The isolates' development were assessed relative to the control treatment (without metals) and classified as tolerant or not tolerant.

## 3. Results

### 3.1. Isolation of Endophytic *Methylobacterium*

**Figure 1:**
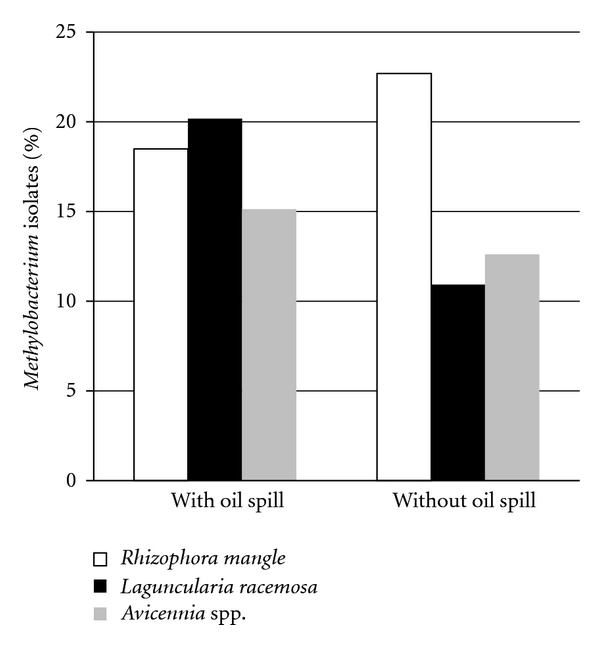
Density of cultured endophytic *Methylobacterium *spp. isolated from mangrove trees in isolated mangrove forests with and without spilled oil in Bertioga, SP, Brazil.

From a total of 716 endophytic bacteria isolated, 109 (15.2%) were pink-pigmented bacteria (Figure 1), making it the most abundant group. To verify that these isolates belonged to the *Methylobacterium *genus, the 16S rRNA gene was amplified with primers specific to this genus, and the gene was amplified in all 109 of the isolates, thereby confirming the bacteria as *Methylobacterium *species. The 109 isolates were selected and tested for tolerance to different concentrations of three heavy metals.

### 3.2. Tolerance to Heavy Metals

**Figure 2:**
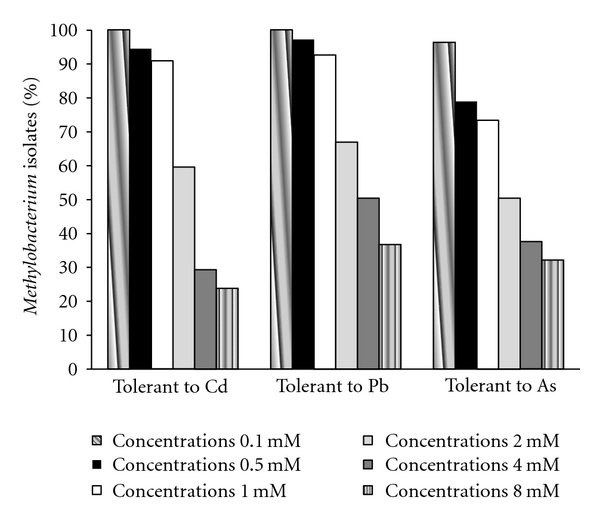
Distribution of endophytic samples isolated from mangrove species of the *Methylobacterium *genus showing tolerance to Cd, Pb, and As at the concentrations 0.1 mM, 0.5 mM, 1 mM, 2 mM, 4 mM, and 8 mM.

There are different metal tolerance mechanisms in bacteria including efflux transport, complexation (intracellular sequestration, absorption, export of chelated compounds), and metallic ion reduction; moreover, the oxidation state of each metal affects its solubility and toxicity [23]. All of the tested metals (Cd(+2), Pb(+2), and As(+5)) were soluble in the culture medium. Of the 109 isolates studied, 70% were tolerant to all three metals at a 1 mM concentration; however, at 8 mM, the highest concentration tested, only five isolates remained tolerant to Cd, 21 isolates exhibited tolerance to Pb, and 21 isolates exhibited tolerance to As. Only two isolates, both isolated from the *Avicennia* spp. at the location without an oil spill (Figure 2), displayed tolerance to all three metals tested. The genetic determinants of resistance to these three metals can be located on plasmids [23–25].

### 3.3. Phylogenetic Diversity of Endophytic *Methylobacterium* spp. in Mangrove Trees

**Figure 3:**
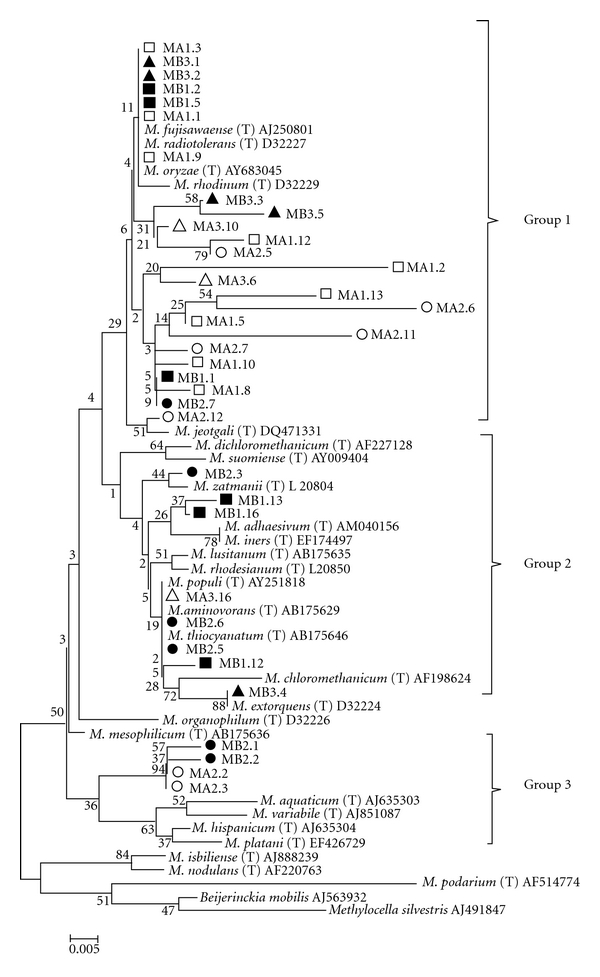
Phylogenetic tree built through sequence analysis of 400 bp of the 16S rRNA gene of 36 of the isolated* Methylobacterium *spp. by the *Neighbor-joining *method. *Beijerinckia *and *Methylocella* were used as outgroups. Bootstrap values higher than 40 are shown on the nodes' intersections (*n* = 1000 replicates). The isolates of the different hosts and origins are marked as follows: *Rhizophora mangle *at the location with an oil spill □, *Rhizophora mangle *at the location without an oil spill ■, *Laguncularia racemosa* at the location with an oil spill *▵*, *Laguncularia racemosa* at the location without an oil spill ▴, *Avicennia *spp. at the location with an oil spill *⚪*, and* Avicennia *spp. at the location without an oil spill *⬤*.

Following the exposure to the heavy metals, 400 bp of the 16S rRNA gene of 36 isolates were sequenced and deposited in the GenBank database under the accession numbers JF422737 to JF422771. A phylogenetic tree representation of these isolates is found in Figure 3.

Most of the isolates were similar to the *M. fujisawaense, M. radiotolerans, *and* M. oryzae *species and were classified into Groups 1, 2, and 3. Groups 1 and 2 numbered endophytes isolated from all three of the plant species studied here. However, Group 3 comprised only four isolates, all from the same host *Avicennia* spp., with two from the location with an oil spill and two from the site without an oil spill. This indicates that there is some specificity in the relationships between bacterial species and host plants.

Some isolates did not show any similarity to any isolates found in the database; however, these may represent *Methylobacterium* species that have not yet been described. Group 2 contained isolates from all three host plants, and all of them were similar to different known *Methylobacterium* species. All but one of these isolates were discovered in the oil-contaminated site.

In addition, this phylogenetic analysis shows that isolates from Group 1 clustered more isolates tolerant to all tested metals (8, 14 and 10 isolates tolerant to 8 mM of Cd, Pb, and As, resp.) (Table 1), confirming that the two isolates that were tolerant to all three metals (MB3.2 and MB3.3) were isolated from the location without an oil spill and were placed in Group 1, an large group that also contained many isolates from the contaminated location that are similar to *M. fujisawaense, M. radiotolerans, *and* M. oryzae *species (Figure 3). While, Group 3 present only 1 isolate tolerant to 8 mM of As (Table 1).

## 4. Discussion

We isolated a diverse group of endophytic microorganisms from samples collected in Bertioga, São Paulo State, Brazil, from locations with and without an oil spill, and from three different mangrove plant species. However, both locations, whether contaminated with oil or not, suffer from anthropogenic influences due to proximity to industrial and domestic sewers, and because of the petroleum spill and sewer runoff, there are heavy metals present in these environments.

The description of the bacterial diversity within the mangrove plant hosts contributes to our understanding of these microorganisms in this ecosystem. This highly adapted bacterial species is a potential source of biotechnological resources for future investigations, such as the search for bacteria that can promote bioremediation of a polluted environment. The endophytes colonize the inner tissues of their plant hosts without causing disease, and they can establish mutualistic associations with the hosts, promoting a better adaptation of the host plants to the environment through mechanisms including the immobilization of heavy metals that are toxic to the plants.

Multiple authors have demonstrated that *Methylobacterium* spp. can be tolerant of to up to 4 mM Cd, 8 mM Pb, and 48 mM As [15, 26]. In this study, most of the isolates (70%) were tolerant to 1 mM Cd, As, and Pb, concentrations higher than would be found in the natural environment (soil and oceans or rivers). Moreover, two isolates tolerated Cd, Pb, and As tested at a concentration of 8 mM. This concentration of Cd tolerated by *Methylobacterium* sp. was higher than that found in any previous studies, presumably due to this soil microorganism becoming an endophyte. Also, Cd is more labile in the soil, allowing the bacteria to become more tolerant to cadmium (plasmid associated) [27]. On the other hand, Pb is not labile in the soil, thus the bacteria are less tolerant [26]. Nevertheless, two isolates showed tolerance to heavy metals in concentrations higher than described previously in the literature (except for As), thus present a potential use in the bioremediation of locations contaminated with heavy metals, such as the mangrove forest with an oil spill or other locations that suffer anthropogenic action due to industrial and domestic sewers. 

We detected endophytic methylotrophic bacteria in the three different species of mangroves. These bacteria displayed differing levels of resistance to heavy metals, from 0.1 mM to 8.0 mM of As, Cd, and Pb. Cadmium is the most selective heavy metal assessed in this work, followed by arsenic and lead. The methodologies used to identify and analyze the genetic diversity of pink-pigmented bacteria showed that the isolates from the forest that had undergone oil spillage were grouped, suggesting that oil can select microorganisms that tolerate or degrade oil compounds and can change the methylotrophic bacterial community accordingly.

The two isolates that are tolerant to cadmium, lead, and arsenic were selected by oil and present a potential for use in bioremediation of this environment by their action in immobilizing these metals. Further study is required to determine the mechanisms of tolerance. These tolerant bacteria are endophytic and present a direct association with plants, strongly suggesting that these bacteria immobilize the metal, promoting plant growth. Therefore, the isolation and characterization of the *Methylobacterium* spp. is the first step to enable the future bioremediation of this area through removal of the heavy metals from of mangrove forest though a reactor or by phytoremediation with endophytic bacteria [28–30].

To our knowledge, this is the first report of the genetic diversity of the *Methylobacterium *spp. community in a mangrove forest that showed tolerance to heavy metals in *in vitro* assays.

## Figures and Tables

**Table 1:** Identification of mangrove endophytic *Methylobacterium* spp. by the partial sequence of the 16S rRNA and its tolerance to all tested metals (Cd, Pb, and As) on the higher tested concentration (8 mM).

	Isolate	Mangrove area	Host	Identification*	Phylogenetic groups	Heavy metals		
						Cd	Pb	As
						8 mM	8 mM	8 mM
1	MA1.1	Oil spill contaminated	*Rhizophora mangle*	*Methylobacterium *sp.	1	−	−	+
2	MA1.2	Oil spill contaminated	*Rhizophora mangle*	*Methylobacterium *sp.	1	+	−	+
3	MA1.3	Oil spill contaminated	*Rhizophora mangle*	*Methylobacterium *sp.	1	+	−	+
4	MA1.5	Oil spill contaminated	*Rhizophora mangle*	*Methylobacterium *sp.	1	+	+	+
5	MA1.8	Oil spill contaminated	*Rhizophora mangle*	*Methylobacterium *sp.	1	−	−	−
6	MA1.9	Oil spill contaminated	*Rhizophora mangle*	*Methylobacterium *sp.	1	−	+	−
7	MA1.10	Oil spill contaminated	*Rhizophora mangle*	*Methylobacterium *sp.	1	+	+	+
8	MA1.12	Oil spill contaminated	*Rhizophora mangle*	*Methylobacterium *sp.	1	−	−	−
9	MA1.13	Oil spill contaminated	*Rhizophora mangle*	*Methylobacterium *sp.	1	−	−	−
10	MA2.2	Oil spill contaminated	*Laguncularia racemosa*	*Methylobacterium *sp.	3	−	−	−
11	MA2.3	Oil spill contaminated	*Laguncularia racemosa*	*Methylobacterium *sp.	3	−	−	+
12	MA2.5	Oil spill contaminated	*Laguncularia racemosa*	*Methylobacterium *sp.	1	+	−	+
13	MA2.6	Oil spill contaminated	*Laguncularia racemosa*	*Methylobacterium *sp.	1	−	−	−
14	MA2.7	Oil spill contaminated	*Laguncularia racemosa*	*Methylobacterium *sp.	1	−	+	−
15	MA2.11	Oil spill contaminated	*Laguncularia racemosa*	*Methylobacterium *sp.	1	−	+	+
16	MA2.12	Oil spill contaminated	*Laguncularia racemosa*	*M. jeotgali*	1	−	−	−
17	MA3.6	Oil spill contaminated	*Avicennia *spp.	*Methylobacterium *sp.	1	−	+	−
18	MA3.10	Oil spill contaminated	*Avicennia *spp.	*Methylobacterium *sp.	1	−	+	−
19	MA3.16	Oil spill contaminated	*Avicennia *spp.	*Methylobacterium *sp.	2	−	−	−
20	MB1.1	Uncontaminated	*Rhizophora mangle*	*Methylobacterium *sp.	1	−	+	−
21	MB1.2	Uncontaminated	*Rhizophora mangle*	*Methylobacterium *sp.	1	+	+	+
22	MB1.5	Uncontaminated	*Rhizophora mangle*	*Methylobacterium *sp.	1	−	−	−
23	MB1.12	Uncontaminated	*Rhizophora mangle*	*Methylobacterium *sp.	2	+	−	−
24	MB1.13	Uncontaminated	*Rhizophora mangle*	*Methylobacterium *sp.	2	−	−	−
25	MB1.16	Uncontaminated	*Rhizophora mangle*	*Methylobacterium *sp.	2	+	−	+
26	MB2.1	Uncontaminated	*Laguncularia racemosa*	*Methylobacterium *sp.	3	−	−	−
27	MB2.2	Uncontaminated	*Laguncularia racemosa*	*Methylobacterium *sp.	3	−	−	−
28	MB2.3	Uncontaminated	*Laguncularia racemosa*	*M. zatmanii*	2	−	−	+
29	MB2.5	Uncontaminated	*Laguncularia racemosa*	*Methylobacterium *sp.	2	−	−	−
30	MB2.6	Uncontaminated	*Laguncularia racemosa*	*Methylobacterium *sp.	2	+	−	+
31	MB2.7	Uncontaminated	*Laguncularia racemosa*	*Methylobacterium *sp.	1	−	−	−
32	MB3.1	Uncontaminated	*Avicennia *spp.	*Methylobacterium *sp.	1	−	+	−
33	MB3.2	Uncontaminated	*Avicennia *spp.	*Methylobacterium *sp.	1	+	+	+
34	MB3.3	Uncontaminated	*Avicennia *spp.	*Methylobacterium *sp.	1	+	+	+
35	MB3.4	Uncontaminated	*Avicennia *spp.	*M. extorquens*	2	+	+	−
36	MB3.5	Uncontaminated	*Avicennia *spp.	*Methylobacterium *sp.	1	−	+	−

*Identification based on the RDP database (http://www.simo.marsci.uga.edu/public_db/rdp_query.htm) and on the phylogenetic analysis in this study (Figure 3).
